# The Redox Communication Network as a Regulator of Metabolism

**DOI:** 10.3389/fphys.2020.567796

**Published:** 2020-10-15

**Authors:** Barbara E. Corkey, Jude T. Deeney

**Affiliations:** Department of Medicine, Boston University School of Medicine, Boston, MA, United States

**Keywords:** redox, ROS, metabolic regulation, network, energy metabolism, β-cells, adipocytes, hepatocytes

## Abstract

Key tissues are dysfunctional in obesity, diabetes, cardiovascular disease, fatty liver and other metabolic diseases. Focus has centered on individual organs as though each was isolated. Attention has been paid to insulin resistance as the key relevant pathosis, particularly insulin receptor signaling. However, many tissues play important roles in synergistically regulating metabolic homeostasis and should be considered part of a network. Our approach identifies redox as an acute regulator of the greater metabolic network. Redox reactions involve the transfer of electrons between two molecules and in this work refer to commonly shared molecules, reflective of energy state, that can readily lose electrons to increase or gain electrons to decrease the oxidation state of molecules including NAD(P), NAD(P)H, and thiols. Metabolism alters such redox molecules to impact metabolic function in many tissues, thus, responding to anabolic and catabolic stimuli appropriately and synergistically. It is also important to consider environmental factors that have arisen or increased in recent decades as putative modifiers of redox and reactive oxygen species (ROS) and thus metabolic state. ROS are highly reactive, controlled by the thiol redox state and influence the function of thousands of proteins. Lactate (L) and pyruvate (P) in cells are present in a ratio of about 10 reflective of the cytosolic NADH to NAD ratio. Equilibrium is maintained in cells because lactate dehydrogenase is highly expressed and near equilibrium. The major source of circulating lactate and pyruvate is muscle, although other tissues also contribute. Acetoacetate (A) is produced primarily by liver mitochondria where β-hydroxybutyrate dehydrogenase is highly expressed, and maintains a ratio of β-hydroxybutyrate (β) to A of about 2, reflective of the mitochondrial NADH to NAD ratio. All four metabolites as well as the thiols, cysteine and glutathione, are transported into and out of cells, due to high expression of relevant transporters. Our model supports regulation of all collaborating metabolic organs through changes in circulating redox metabolites, regardless of whether change was initiated exogenously or by a single organ. Validation of these predictions suggests novel ways to understand function by monitoring and impacting redox state.

## Why Is a Network Perspective Important?

### Metabolic Changes Must Be Communicated

Traditional molecular and biochemical studies have focused on interactive processes involved in metabolic pathway fluxes in specific cells and organs. Elegant studies have provided important and detailed mechanistic evaluation of the pathways and proteins involved in metabolic regulation, documented changes in protein and RNA expression and identified important relationships, mainly at single points in time. These studies continue to provide detailed molecular mechanisms for metabolic pathway regulation and transcriptional control of metabolic functions. Modern unbiased omics technologies are poised to provide coordinated detailed information about genetic, proteomic, and metabolomic differences among large groups of individuals with specific identifying characteristics, diseases or treatments mainly at single moments in time. These data are potentially useful as markers of disease or treatment efficacy and to draw attention to biological pathways that are unexpected or different among different groups. Such correlations provide a sound basis for generating testable hypotheses regarding causation. However, neither the traditional nor the more modern approaches address the issue of how changes in metabolism are driven and communicated throughout the organism on a variety of time scales, including minute to minute regulation throughout the day.

### Redox, an Energy-Sensitive Communication System

The relevance of neural and hormonal communication networks is well-established and essential for physiological function. However, it is also important that all tissues in the body are aware of the metabolic state and respond rapidly and appropriately to the metabolic energy status in order to maintain the continuous energy supply required for each to function optimally. It is also essential to understand the temporal patterns of change, their linkage and interaction, and often parallel or redundant routes, for achieving similar end results. This article will focus on redox as an initiator of metabolic change that provides communication systems that we hypothesize provides the critical link between several well-established steady-states and the driving force to acutely transition among steady states. Redox reactions involve the transfer of electrons between two molecules and in this work refer to commonly shared molecules that readily lose electrons, to increase, or gain electrons to decrease the oxidation state of molecules including NAD, NADH, NADP, NADPH, and the thiols in their reduced (SH) and oxidized (SS) forms. Metabolism alters all of these redox reactants both rapidly and transiently as well as in various long-term steady-states. Redox reactants comprise an energy-sensitive communication system within each cell and within cellular compartments. Variations in metabolic state can also impact the response of tissues to other communication network systems.

### Redox Systems Adapt to Metabolic Change

Important systems, such as energy synthesis, are often redundant and usually have spare capacity, though they rarely operate at capacity: ATP is produced from ADP by both glycolysis and oxidative phosphorylation, and often both when demand is high as well as through adenylate kinase (Panayiotou et al., 2014). None of these pathways operate near capacity most of the time (Mookerjee et al., 2016). Mitochondria generally respire at 20–30% of capacity and increase or decrease their number and specific enzymes to adapt to high or low demand and fuel availability rather than increasing the percentage of their operating flux capacity. Skeletal muscle mitochondrial biogenesis, morphological changes, and increases in respiratory complex formation are triggered by exercise or need (Menshikova et al., 2006), for example. Obese humans have up to 5.0-fold higher maximal respiratory rates in liver mitochondria than lean persons and patients with fatty liver disease have higher mitochondrial mass, but lower maximal respiration, less well-coupled mitochondria and a greater proton leak (Koliaki et al., 2015). Thus, neither the capacity nor the protein levels of components of these pathways are generally rate-limiting even though increased usage frequently leads to increases in expression of key proteins (Mookerjee et al., 2016). Such alterations in spare capacity are protective of the network and rarely due to the inability of cells to maintain energy supplies. A common example of such time-dependent adaptation is the response of mitochondria to a switch from low fat to high fat in the diet (Iossa et al., 2003; Turner et al., 2007). The diet-induced obesity model most frequently used is the C57Bl6J mouse model (Fergusson et al., 2014) that may be highly responsive to overfeeding, due in part to the lack of the mitochondrial enzyme, nicotinamide nucleotide transhydrogenase (NNT), needed to effectively scavenge ROS (Fisher-Wellman and Neufer, 2012). ROS rises in response to high rates of β-oxidation (Quijano et al., 2016) and this mouse model may reflect a defect in redox/ROS regulation with unknown relevance to human disease. Thus, redox systems adapt and respond to changes in energy source and demand. Changes in mitochondrial content and expression of mitochondrial proteins are most often adaptations to such altered environmental signals.

The energy needs of individual organs must all be met at the same time and at all times in an optimally functioning organism. These include the continuous energy needs of heart and brain, the meal-induced energy needs for nutrient storage and processing by adipose tissue, gut and liver, the brief secretory responses of pancreas and other secretory organs, and the maximum energy needs of exercising muscle. Such highly varied demands, with only occasional nutrient intake, require a competent and rapid information sharing response system.

## What Comprises the Redox Communication System?

### Redox Components

**FIGURE 1:**
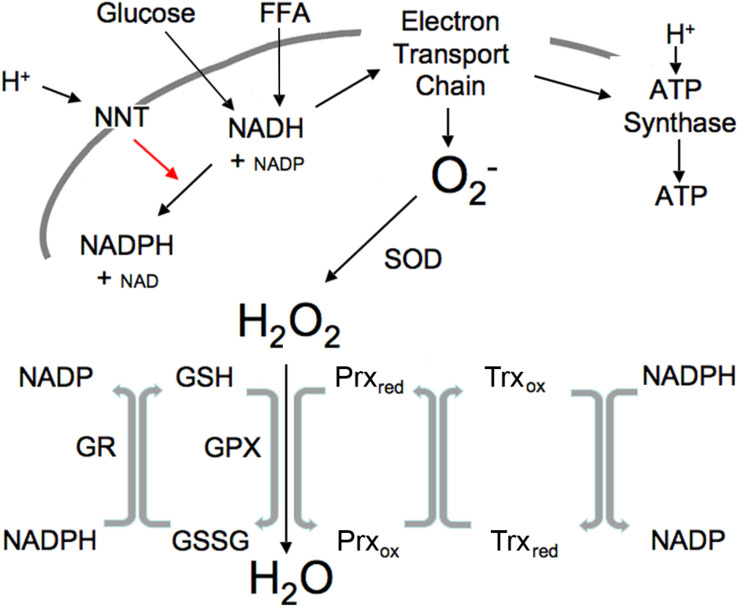
Illustration of mitochondrial interactions among pyridine nucleotides, ROS and the thiol redox system. SOD, superoxide dismutase; NNT, nicotinamide nucleotide transhydrogenase; GPX, GSH peroxidase; Prx, peroxiredoxin; Trx, thioredoxin; R, reductase. Mitochondrial ROS is produced at high NADH levels. NADH is derived from available fuels and promotes ROS production when cellular ATP levels are sufficient. NNT plays a vital role in ROS removal driven by the proton gradient as indicated by red arrow.

Redox components mediate the transfer of electrons between reduced and oxidized compounds. There are several major redox participants that reflect redox state (Jones and Sies, 2015): NAD^+^, NADP^+^, oxidized glutathione (GSSG), thioredoxin (Trx_*ox*_) and peroxiredoxin (Prx_*ox*_) in the oxidized forms, and NADH, NADPH, glutathione (GSH), Trx and Prx, in the reduced form (Figure 1). These redox components are compartmentalized with separate mitochondrial and cytosolic compartments that differ greatly in their redox potential (Jones and Go, 2010). NADH levels are relatively high in the mitochondria in order to provide the electrons or driving force to maintain ATP levels via oxidative phosphorylation, maintain a highly negative membrane potential and to generate the NADPH essential for ROS clearance, whereas NADH levels are relatively low in the cytosol to prevent inhibition of glycolysis (Table 1). In contrast, NADPH levels are high in the cytosol to facilitate synthetic reactions such as *de novo* lipid synthesis as well as functioning to maintain adequate antioxidant defense. The thiol couples are maintained in a reduced state in both compartments in order to protect against oxidative stress (Veech, 2006).

**TABLE 1:** Illustration of Diet-Induced Changes in the NAD/NADH Ratio in Rat Liver [data from Veech (2006)].

**Diet**	**NAD/NADH_cytosol_**	**NAD/NADH_mitochondria_**
Chow	1164	7.7
Fasted-48 h	564	5.5
High Sucrose-72 h	1820	5.1
Low Carb-72 h	526	4.1

### Illustration of Mitochondrial ROS Production

Reactive oxygen species is produced in the mitochondrial electron transport chain when ATP levels are sufficient and excess fuel generates elevated NADH. ROS, generated under these excess fuel conditions, serves as a transient signal of plenty that can be communicated within and outside the cell. Increased ROS subsequently activates the ROS scavenging reactions (Figure 1). This occurs appropriately when substrate availability is high (high NADH) but ATP needs have been fulfilled. Excessive mitochondrial ROS generation can result in H_2_O_2_ release from the cell into the circulation as demonstrated by Oshino and Chance using the perfused liver (Oshino et al., 1975). In their study, increasing the redox state of mitochondrial electron carriers by either high fatty acids or antimycin A increased the rate of H_2_O_2_ production up to four times the endogenous rate. It should be noted that external environmental influences and cytosolic enzymes can also lead to ROS generation (Oshino et al., 1975).

### NNT

Nicotinamide nucleotide transhydrogenase provides an important link between pyridine nucleotide generation by glucose and FFA, ROS and the thiol redox state (Figure 1). Currently available tools are not adequate to measure the variety of specific ROS species in real-time, hence ROS is usually measured as H_2_O_2,_ the most stable product of superoxide dismutase. NNT (Hoek and Rydstrom, 1988) is a ROS-scavenging enzyme driven by the proton gradient to convert mitochondrial NADH plus NADP to the NADPH needed to maintain thiols in the reduced state by converting oxidized thiols to their reduced form, thus permitting reduced thiols to convert H_2_O_2_ to H_2_O (Figure 1).

### An Intracellular-Redox Communication System Among Pyridine Nucleotides, ROS and Thiols

The interactions among the pyridine nucleotides, ROS and thiols allow changes in one to impact the redox state of the others (Figure 1). ROS levels are usually tightly controlled through NNT and the activity of peroxidases that convert ROS to water as also illustrated in Figure 1. Metabolizing ROS to water oxidizes GSH. NADPH is required to restore resultant GSSG to active GSH. Thus, increases in ROS lead to thiol oxidation that in turn requires NADPH to maintain the pool of active state thiols. In the mitochondria, NADPH is derived largely from NADH through the activity of the NNT. Under basal conditions when fuel is plentiful and ATP demand minimal, NADH levels and the NADH/NAD ratio are high, ROS production is increased, however, the fuel supply is sufficient to provide the needed NADPH to maintain thiols in the reduced state and convert the ROS to water. Since NNT is a transmembrane protein that uses the mitochondrial proton gradient to drive the interconversion of NADH and NADPH, NADH is also required to restore the mitochondrial membrane potential that is used to drive NNT. This may explain a portion of the mitochondrial proton leak that is greater under basal than stimulated conditions when ROS is higher. Although NNT is not the only mitochondrial source of NADPH, it is a major component (Ronchi et al., 2016). A separate but analogous antioxidant system exists in the cytosol where the major sources of NADPH are the pentose phosphate pathway, malic enzyme and isocitrate dehydrogenase. These systems that produce and scavenge ROS, provide rapid and transient communication of nutrient availability through interactions among all of the redox components.

## How Is Energy State Communicated?

### Shared Cofactors Within Cells

**FIGURE 2:**
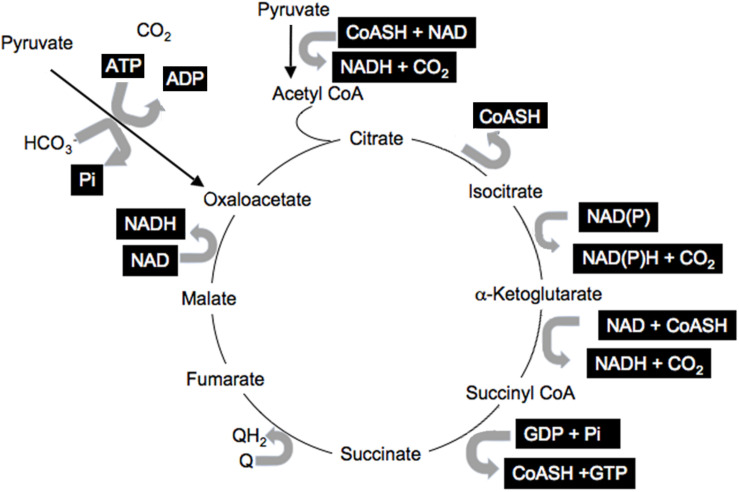
Shared citric acid cycle cofactors. Shared factors are highlighted.

Information within cells is shared via the common co-factors, just described, that are used by many enzymes and pathways (Corkey and Shirihai, 2012). These include pyridine nucleotides, adenine nucleotides, Coenzyme A esters, thiols such as GSH, Prx, and Trx, and ROS. This common currency is used for many enzymatic reactions involving pyridine nucleotides: NAD(P) and NAD(P)H; adenine nucleotides: ATP, ADP, AMP; coenzyme A derivatives: free CoASH, acetyl CoA, long-chain acyl CoA (LC-CoA); while ROS and thiols modulate the many reactive cysteines in proteins and impact translational activity (Marsboom and Rehman, 2016; Hopkins and Neumann, 2019). All participate in numerous reactions in all cells and cellular compartments. A simple illustration of the many reactions involving this shared currency is the citric acid cycle that includes multiple reactions with many shared common co-factors (Figure 2, highlighted co-factors). It is important to note that most of the metabolites and cofactors present in cells do not freely traverse cellular membranes but remained localized in their relevant compartments. Thus, additional mechanisms must be considered that permit sharing of the redox state among cellular compartments and into the circulation.

### Shared Cofactors That Circulate in the Blood

**FIGURE 3:**
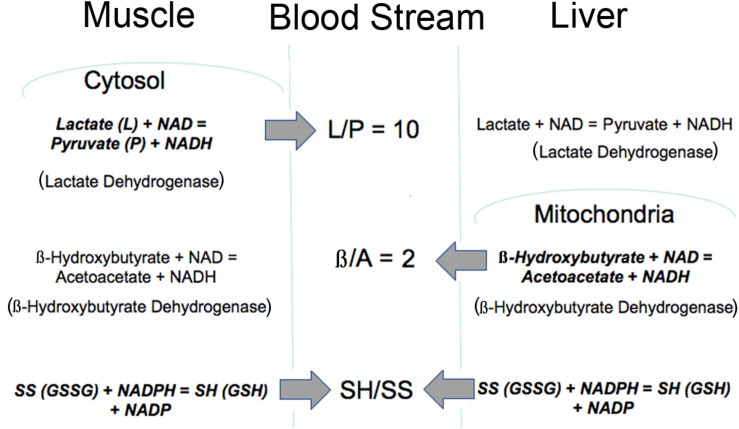
Illustration of how the intracellular redox state is communicated to the blood stream via common metabolites. The thiol ratio is reflected mainly in the cysteine to cystine ratio but also in the GSH/GSSG ratio. The mitochondrial pyridine nucleotide redox state is reflected in the β/A ratio and regulated mainly by liver mitochondria. The cytosolic pyridine nucleotide redox state is reflected in the L/P ratio and regulated mainly by muscle. All of these redox indicators are readily transported into and out of cells. Bold type indicates reaction is mainly regulated in that tissue.

Importantly, the mitochondrial metabolic state can be known through blood metabolites that are shared among different organs via circulating communication metabolites. These indicator metabolites readily traverse mitochondrial and plasma membranes out of the cell of origin and into destination cells where they impact the use of shared cofactors and reactions involving those shared cofactors, as previously illustrated (Figures 1, 2). Abundant membrane transporters are key to allowing specific metabolites to enter and leave cells rapidly, according to their concentration gradient, moving from higher to lower concentration compartments (Figure 3). Specific substrate-product pairs are in equilibrium with NAD and NADH in their respective compartments. Pyruvate (P) and lactate (L) are in equilibrium with a high NAD/NADH ratio (low NADH) driven by lactate dehydrogenase in the cytosol whereas acetoacetate (A) and β-hydroxybutyrate (β) are in equilibrium with a low NAD/NADH ratio (high NADH) driven by β-hydroxybutyrate dehydrogenase in the mitochondria. Because information about the cytosolic and mitochondrial redox states are reflected in the circulation through these common metabolites, this information is communicated from the cell of origin to other cells accessed by the circulation. As a consequence, the muscle cytosolic redox state, reflected in the L/P ratio, has the greatest impact on cytosolic redox throughout the system because of the large muscle mass. On the other hand, the liver mitochondrial redox state is a dominant influence on the mitochondria of other tissues because of its greater capacity to oxidize fat and to produce, but importantly, not use, the ketones, acetoacetate and β-hydroxybutyrate (Cahill, 1971). Thus, the pyridine nucleotide redox couples communicate directly in both directions between the cell and the circulation. The thiol redox state is regulated and communicated differently (Moriarty-Craige and Jones, 2004). Intracellular levels of GSH are in the high mM range and highly reduced in both the cytosol and mitochondria, while cysteine levels are lower in the range of 10–50 μM, and also reduced. Cysteine and cystine mainly function as precursors to GSH and other synthetic peptides and proteins within cells. In contrast, in plasma the reverse is true and cystine levels are present at higher concentrations, highly oxidized and the main indicator of an increased oxidation state due in part to the oxidizing impact of the plasma on thiols (Turell et al., 2013). The thiol transport systems are less well characterized although the transport of all 4 thiols is well-documented, however, the cellular thiol redox state is not directly reflected in the plasma thiol redox state. The time course of change in the blood thiol redox state has not been documented *in vivo* and may be slower than the pyridine nucleotide redox state. Cystine is readily imported into cells where it is converted to cysteine, the limiting precursor for GSH formation (Yin et al., 2016). GSH is mainly formed in the cytosol, however, GSSG is transported out of cells particularly when elevated possibly to maintain the high GSH/GSSG ratio although GSH is also transported out of liver cells possibly as a source of cysteine for other cells (Oestreicher and Morgan, 2019). Thus, changes in the cellular thiol redox state are best reflected in the plasma cysteine/cystine ratio despite the magnitudes of difference in their actual electrochemical potential (Go and Jones, 2013a,b) while changes in the intracellular pyridine nucleotide redox state are directly reflected in their interacting circulating metabolites. Thus, different tissues make distinct contributions to circulating redox levels that together communicate the metabolic state of the body.

## Induction of Changes in Intracellular ROS and Function in Response to Physiological Variations in Extracellular Redox Couples

A redox communication system that shares information among metabolically sensitive tissues predicts that physiologically relevant changes in the putative redox indicator ratios will impact tissue ROS production and function.

### Oxidized Thiols and Disease

In the case of the thiol redox state, many publications by D. P. Jones and colleagues have shown that thiols become oxidized in many disease states. Oxidation affects cell proliferation, apoptosis, and proinflammatory signaling. Such effects have been observed in endothelial cells, fibroblasts, monocytes, and epithelial cells, with cell-specific responses (Go and Jones, 2010a). Both circulating GSH and cysteine systems become oxidized with aging, and a recent finding suggests that the cystine to GSH ratio in human plasma predicts the likelihood of death in coronary artery disease (Jones, 2015). Retinal pigment epithelial cells exposed to a more oxidized extracellular redox environment have increased susceptibility to oxidant-induced apoptosis (Jiang et al., 2005). Other results suggest a prominent role for the extracellular thiol redox status in regulation of cell invasion (Chaiswing et al., 2008).

Go and Jones (2010a) developed a technique to study intracellular signaling in response to extracellular redox potential changes, using a redox clamp in which thiol and disulfide concentrations are varied to obtain a series of controlled redox potentials. We applied these techniques using the thiols as well as the β/A and L/P ratios to further assess the impact of physiological changes in extracellular redox on function in human adipocytes and isolated mouse hepatocytes.

### Hepatic ROS Production

Changes in ROS production occur in response to variations in pyridine nucleotide couples over a physiological range of electrochemical potentials (Nocito et al., 2015). As the thiol and β/A ratios become more oxidized, ROS production increases. It is noteworthy that the cytosolic redox couple, L/P, decreases ROS production as it becomes more oxidized, the opposite effect to changes induced by the thiol and mitochondrial redox couples. This is presumably due to the dominant ability of pyruvate to enter the mitochondria and increase NADH, although it has the opposite oxidizing effect in the cytosol. Thus, the lactate to pyruvate ratio effectively reports the cytosolic redox state but changes in external lactate and pyruvate cannot be used to change the cytosolic ratio without also impacting the mitochondrial redox state in the opposite direction.

In response to redox-induced ROS production, gluconeogenesis is inhibited over a physiological range of electrochemical potentials (Nocito et al., 2015). Since high ROS that accompanies the more oxidized state is normally an indicator of fuel excess, it is logical and appropriate that glucose production by the liver should be inhibited under these conditions. High ROS is usually a transient indicator of fuel excess that stimulates ROS scavenging using NADPH derived from NADH in the presence of active ROS scavenging capacity. Inadequate scavenging capacity, insufficient NADPH production or excessive ROS generation has highly detrimental consequences leading to uncontrolled oxidative stress (Ryter et al., 2007).

### Adipocyte ROS Generation

Similar responses to variations in extracellular redox potential are also observed in adipocytes (Jones et al., 2016) and may be anticipated in many other cell types. Lipolysis requires ROS and is stimulated by the more oxidized, ROS-generating extracellular redox couples. Addition of the ROS scavengers, DPI, NAC or resveratrol, inhibit lipolysis under all conditions tested. At the same time ROS is also required for triglyceride synthesis. ROS removal with the ROS scavenger, DPI, blocks lipid synthesis in fat cells whose primary function is to store fat by this pathway. Thus, both lipid synthesis and breakdown require ROS and increase in response to increasing ROS production. Quantitation of the relative changes and comparison of the concentration dependence of ROS-mediated stimulation of lipid synthesis compared with lipolysis have not yet been determined.

## When Does the Circulating Redox State Change?

### Circulating Redox Changes

Circulating redox changes occur in response to fasting and following a meal (Williamson et al., 1967). A more oxidized state is observed in obese compared with lean subjects or in response to high fat feeding (Anderson et al., 2009a,b). During a 24 hr fast in humans as illustrated in Table 2, increases occur in the ketones, acetoacetate plus β-hydroxybutyrate, from less than 0.1 mM to more than 7 mM. These ketones are formed from FFA that also increase from about 0.7 mM to about 1.6 mM (Cahill, 2006). Changes in the β/A ratio also occur but do not correlate with total ketones since they reflect NADH availability in the mitochondria (Veech, 2006) rather than total ketones. In the illustration in Table 2, the increase in ketones is greater than 70-fold whereas the β/A ratio only increases 2.3-fold.

**TABLE 2:** The Effect of 24 h Fasting on Ketones, Redox, and FFA [data from Cahill (2006)].

**Hours**	**Ketones (mM)**	**β/A Ratio**	**FFA (mM)**
Meal (*t* = 0)	0.09	2.0	0.66
3	0.10	2.3	0.71
6	1.62	3.0	1.25
24	7.05	4.6	1.55

### Blood Redox Metabolites Change Acutely as Illustrated in a Patient Fasted Overnight Then Fed High Glucose

**FIGURE 4:**
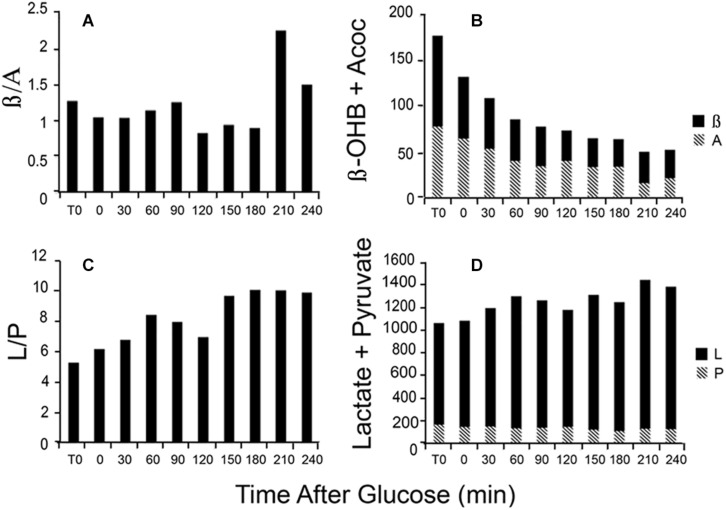
Illustration of the time course of changes in blood metabolites in response to a glucose load. Data are from a single patient. **(A)** The β/A ratio; **(B)** the sum of β plus A; **(C)** the L/P ratio; and **(D)** the sum of L plus P. Assays were performed on neutralized acid extracts, prepared rapidly after blood samples were taken, and analyzed within 24 h (Williamson and Corkey, 1969, 1979).

Changes in the L/P ratio, lactate plus pyruvate, the β/A ratio, and total ketones occur rapidly in response to glucose feeding. Figure 4 illustrates data obtained from a single patient in our clinic. During the first hour, total ketones decrease rapidly as glucose replaces FFA as the main fuel source while lactate rises modestly as glucose, in excess of energy needs, is metabolized to lactate. The β/A ratio decreases slightly reflecting increased energy use but remains rather constant for the first 3 h while the L/P ratio gradually increases as fuel needs are met. The thiol redox state remains fairly constant (data not shown). These data illustrate that rapid responses to nutrient intake can be observed through the blood redox metabolome, in a time frame of minutes. These changes are consistent with expected variations in energy state and fuel use.

### Changes in Aging and Disease States

As elegantly documented by Dean Jones over many years (Jones, 2002; Moriarty-Craige and Jones, 2004; Go et al., 2009; Adimora et al., 2010; Go and Jones, 2010b, 2013a,b), the blood thiol redox state becomes more oxidized in diabetes, aging and cancer, presumably due at least partially to excessive ROS production or inadequate ROS scavenging. These changes are commonly viewed as markers of oxidative stress. ROS production also increases in response to excess fuels, as described in section “Redox components,” although the acute changes observed in pyridine nucleotide redox state in our patient were not reflected in marked changes in the thiol redox state during the 6 h procedure.

### Regulation of Hepatic Redox State

**FIGURE 5:**
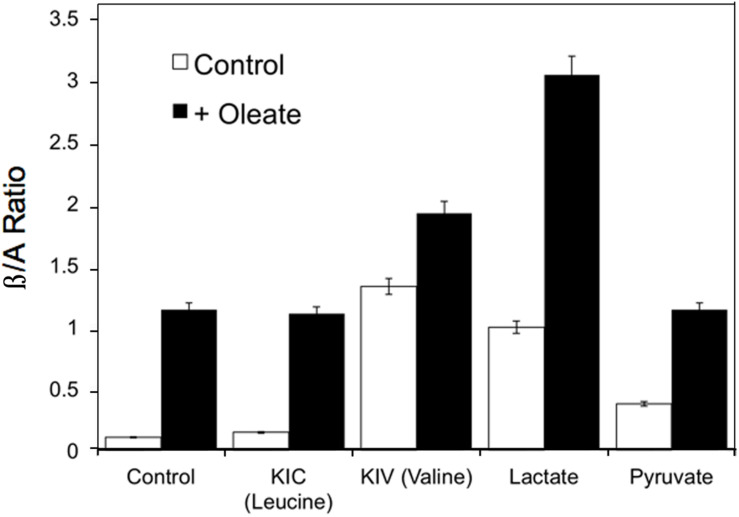
Example of the changes in β/A ratios in isolated hepatocytes in response to the ketoacids of leucine, valine and isoleucine, carbohydrate- derived, lactate and pyruvate, and the FFA, oleate. Data derived from references (Corkey et al., 1981, 1982).

Reactive oxygen species also increases in response to excess fuels, as described in section “Redox Components” in perfused liver: lactate plus pyruvate and FFA stimulate ROS production. Antimycin A, which inhibits the electron transport chain after the ROS generating step, further increases ROS production in the presence of excess fuel (Boveris et al., 1972) due in part to reverse electron flow. Mitochondrial redox state changes in liver in response to fasting, high sucrose and low carbohydrate feeding (Veech et al., 1969). In response to fasting and low carbohydrate feeding cytosolic and mitochondrial NADH increase while mitochondrial NADPH decreases. In contrast, sucrose feeding decreases cytosolic while increasing mitochondrial NADH with little or no effect on NADPH (Veech et al., 1969). Generally, increases in mitochondrial NADH occur in response to fuel availability within hepatocytes whether the fuel is fat, amino acid or carbohydrate. Figure 5 illustrates the increases in β/A ratios in isolated hepatocytes in response to the amino acids, leucine, valine and isoleucine, carbohydrate-derived lactate and pyruvate, as well as the FFA, oleate (Corkey et al., 1981, 1982). These hepatic mitochondrial changes can be communicated throughout the organism via the blood stream by the metabolites that circulate: β-hydroxybutyrate and acetoacetate. Note that although only FFA and leucine form ketones, the addition of other fuels that do not form ketones can, however, alter the β/A ratio. This occurs because all fuels produce NADH, which enters a common pool that can donate electrons to the electron transport chain to drive ATP production and also impact the β/A ratio via β-hydroxybutyrate dehydrogenase.

## Environmental Factors Impact β-Cells and Adipocytes

### The Altered Metabolic Environment

Environmental agents, known to cause oxidative stress, can also increase ROS and insulin secretion in the absence of a stimulatory fuel (Simmons et al., 2014). Many environmental changes have accompanied the current epidemic of obesity and diabetes. Much has changed in our world that might explain this epidemic, however, many of the changes have not yet been carefully studied. Our foods have changed, living conditions, activity levels, the air we breathe have all changed. It is important to consider the possibility that redox changes, similar to those that occur in response to nutrients, may also be induced by some food additives and may thus serve to mis-communicate the metabolic status to all tissues (Mangge et al., 2013; Chassaing et al., 2015; Laster and Frame, 2019). Such redox changes influence tissue specific functions at least in part through generation of ROS, which is normally an indicator of fuel sufficiency. The possibility that environmental impacts may lead to changes in circulating redox, is potentially an important and unrecognized form of inter-organ communication. More detailed investigations on pancreatic β-cell insulin secretion support such possibilities (Corkey, 2012a,b; Berdan et al., 2016; Erion and Corkey, 2018).

### Bisphenol A, Contained in Plastics

The identification of endocrine disrupting chemicals or obesogens is a rapidly evolving field of research (Heindel and Blumberg, 2019). BPA is one of the most prevalent chemicals in our environment that leaches from plastic bottles and BPA-lined cans (Simmons et al., 2014). Strong positive correlations have been reported between urine BPA concentration and BMI (Elobeid and Allison, 2008). *In vitro* and *in vivo* studies have shown that BPA accelerates adipocyte differentiation and promotes lipid accumulation via alteration of glucose homeostasis (Wang et al., 2012). In addition, BPA has been shown to increase ROS production in blood and sperm cells (Silveira et al., 2019).

### H_2_O_2_ Directly and Indirectly Increases Insulin Secretion in β-Cells

Increases in H_2_O_2_, like excess fuel, stimulate insulin secretion but in the absence of a stimulatory fuel (Pi et al., 2007, 2010). Our studies document stimulation of insulin secretion by low concentrations of H_2_O_2_, whether added to the outside of β-cells or generated within, without any change in glucose or other fuel concentration. In contrast, provision of ROS scavengers, such as cell permeant catalase or N-acetyl-L-cysteine, inhibit both glucose-stimulated H_2_O_2_ accumulation and insulin secretion. Furthermore, acute exposure of isolated mouse islets or INS-1(832/13) β-cells to the oxidative stressors, arsenite, 4-hydroxynonenal, or methylglyoxal, decreased insulin secretion (Adimora et al., 2010; Go and Jones, 2010b).

**FIGURE 6:**
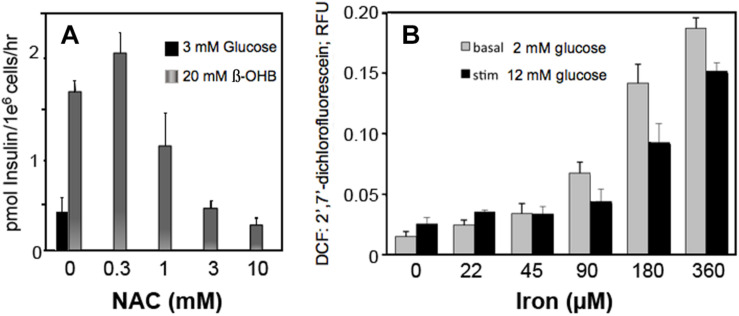
Effect of β-hydroxybutyrate (β-OHB) **(A)**, Iron **(B)** and ROS removal **(A)** on stimulation of insulin secretion **(A)** and ROS generation **(B)** in clonal pancreatic β-cells (Adimora et al., 2010; Go and Jones, 2010b). **Panel A** shows insulin secretion at basal glucose in response to 20 mM β-OHB and concentration-dependent reversal with increasing NAC (n-acetyl cysteine). **Panel B** shows concentration-dependent ROS production from iron at basal and stimulatory glucose.

Circulating metabolites, like β-hydroxybutyrate that enter cells and increase NADH and ROS production in β-cells, increase insulin secretion (Figure 6A). Scavenging ROS with N-acetylcysteine inhibits insulin secretion by decreasing ROS (Figure 6A). These findings suggest that H_2_O_2_, whether derived from glucose metabolism or exogenous sources, is a sufficient and essential metabolic signal for insulin secretion.

### Iron, an Essential Mineral

Iron consumption has increased as the lean content of food animals has increased in response to nutritional recommendations for decreased fatty foods. Our data show that iron increased ROS production in β-cells at both low and high glucose (Figure 6B).

### Mono-Oleoyl Glyceride (MOG) Stimulates Basal Insulin Secretion

Mono-oleoyl glyceride, is a natural product and a common additive to most dairy products, which is used as preservative and emulsifier that prevents cream from separating. ROS is generated when MOG is added to unstimulated β-cells (Saadeh et al., 2012; Berdan et al., 2016). MOG also increases the mitochondrial redox state and interestingly, basal insulin secretion, while ROS scavengers abrogate secretion. It was not determined precisely whether MOG or a compound derived from MOG was responsible for the increases in ROS since MOG is readily metabolized to glycerol and LC-CoA (Saadeh et al., 2012; Berdan et al., 2016).

### Saccharin Increases ROS in β-Cells and Fat Cells

**FIGURE 7:**
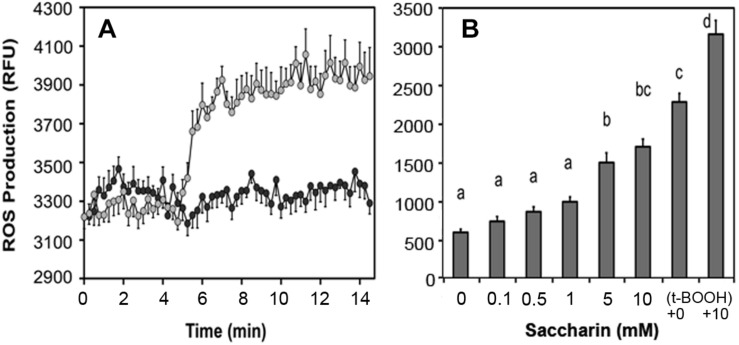
Effect of saccharin on ROS production in clonal pancreatic β-cells **(A)** and human adipocytes **(B)**. A, Dark circles are vehicle, light circles are 5 mM saccharin. B, Concentration-dependent ROS production in cultured, differentiated human adipocytes in response to increasing concentrations of saccharin. The positive controls are tert-butyl hydroperoxide (t-BOOH) with and without 10 mM saccharin.

Artificial sweeteners affect insulin secretion in rat islets: All sweeteners tested generated ROS and increased insulin secretion at low non-stimulatory glucose. We observed that saccharin, sucralose, and aspartame elevated basal insulin secretion in rat islets (Corkey, 2012a,b) and increased ROS production (Figure 7A). In human adipocytes, saccharin increased ROS production at concentrations between 0.1 and 10 mM (Figure 7B). Also, addition of saccharin early in the adipocyte differentiation process promoted lipid accumulation. Saccharin-induced effects in β-cells and adipocytes were largely overcome using antioxidants implicating a ROS-related mechanism.

## Summary and Implications

Coordination of many processes is essential to fulfill the energy needs of all cells on a continuing basis. This article describes a redox network that can share information and elicit responses to that information in a coordinated manner throughout the entire body. Support for such a network involves specific intracellular shared indicators of metabolic state and describes how they interact with the circulation and how the circulation shares this information throughout the system. The specific redox couples, which are readily transported into and out of most cells, are present in the circulation and can interact with all cells that express metabolite transporters. Examples of physiological conditions where redox changes are well-documented and possible causes of environmentally-induced misinformation suggested. Documentation of a redox communication system has been demonstrated in β-cells, adipocytes and liver. ROS and redox changes occur rapidly and frequently and impact function in an organ-specific manner. The model presented here, introduces the novel concept of redox as a master early regulator of metabolism that may be the initiating step in modulating transcription and altering protein expression. This is perhaps analogous to the generally accepted concept of transcriptional master switches that regulate families of anabolic and catabolic genes. It would be interesting to assess the consequences of altered redox state on transcriptional regulation as this has not yet been rigorously investigated. Data also suggest that it is important to consider environmental factors that have arisen coincident with the current epidemic of metabolic dysfunction as potential modifiers of redox or ROS and purveyors of possible misinformation and inappropriate adaptation.

## Author Contributions

BC obtained funding, conceived and designed experiments, analyzed data, proposed model, and wrote the manuscript. JD conceived, designed and performed experiments, analyzed data, and edited the manuscript. Both authors contributed to the article and approved the submitted version.

## Conflict of Interest

The authors declare that the research was conducted in the absence of any commercial or financial relationships that could be construed as a potential conflict of interest.
